# Maresin1 alleviates liver ischemia/reperfusion injury by reducing liver macrophage pyroptosis

**DOI:** 10.1186/s12967-023-04327-9

**Published:** 2023-07-16

**Authors:** Tong Li, Houshuai Zeng, Wenjing Xian, Hongxing Cai, Jianbo Zhang, Shiji Zhou, Yingxue Yang, Min Luo, Peng Zhu

**Affiliations:** 1 Department of Gastrointestinal Surgery, The Second Affiliated Hospital of Chongqing Medical University, 74 Linjiang Road, Yuzhong District, Chongqing, 400010 China; 2 Department of Anesthesiology, The First Affiliated Hospital of Chongqing Medical University, Chongqing, China; 3 Department of Gastroenterology, The Second Affiliated Hospital of Chongqing Medical University, Chongqing, China; 4 Department of Infectious Diseases, The Second Affiliated Hospital of Chongqing Medical University, Chongqing, China

**Keywords:** Maresin1, Ischemia/reperfusion, Liver, Mitochondria, Pyroptosis

## Abstract

**Background:**

Cell pyroptosis has a strong proinflammatory effect, but it is unclear whether pyroptosis of liver macrophages exacerbates liver tissue damage during liver ischemia‒reperfusion (I/R) injury. Maresin1 (MaR1) has a strong anti-inflammatory effect, and whether it can suppress liver macrophage pyroptosis needs further study.

**Methods:**

This study aimed to investigate whether MaR1 can alleviate liver I/R injury by inhibiting macrophage pyroptosis. The effects of MaR1 on cell pyroptosis and mitochondrial damage were studied by dividing cells into control, hypoxia/reoxygenation, and hypoxia/reoxygenation + MaR1 groups. Knocking out RORa was used to study the mechanism by which MaR1 exert its protective effects. Transcriptome analysis, qRT‒PCR and Western blotting were used to analyze gene expression. Untargeted metabolomics techniques were used to analyze metabolite profiles in mice. Flow cytometry was used to assess cell death and mitochondrial damage.

**Results:**

We first found that MaR1 significantly reduced liver I/R injury. We observed that MaR1 decreased liver I/R injury by inhibiting liver macrophage pyroptosis. Then, we discovered that MaR1 promotes mitochondrial oxidative phosphorylation, increases the synthesis of ATP, reduces the generation of ROS, decreases the impairment of mitochondrial membrane potential and inhibits the opening of mitochondrial membrane permeability transition pores. MaR1 inhibits liver macrophage pyroptosis by protecting mitochondria. Finally, we found that MaR1 exerts mitochondrial protective effects through activation of its nuclear receptor RORa and the PI3K/AKT signaling pathway.

**Conclusions:**

During liver I/R injury, MaR1 can reduce liver macrophage pyroptosis by reducing mitochondrial damage, thereby reducing liver damage.

**Supplementary Information:**

The online version contains supplementary material available at 10.1186/s12967-023-04327-9.

## Introduction

Although there have been significant advances in the study of liver ischemia‒reperfusion (I/R) injury, it remains one of the main causes of death after liver resection or transplantation [1, 2]. Further reducing liver I/R injury has great clinical significance. As an uncontrolled sterile inflammatory response during liver I/R injury is the main cause of organ damage, finding new mechanisms or important driving factors that cause and exacerbate this inflammatory response thus helps to control liver I/R injury from a new perspective.

Pyroptosis is a recently discovered form of programmed cell death that is characterized by cell membrane perforation and the simultaneous release of a large number of proinflammatory factors [3, 4]. Moderate pyroptosis can help limit the spread of pathogens, but excessive pyroptosis can cause a systemic inflammatory response and tissue damage [5]. In recent years, researchers have found macrophage pyroptosis during I/R injuries in the heart, brain, and kidneys. Liver macrophages account for approximately 15% of all liver cells; thus, liver macrophage pyroptosis has great potential to cause severe inflammatory injuries. However, the specific role and mechanism of macrophage pyroptosis in liver I/R injury are not yet clear.

Maresin1 (MaR1) is a newly discovered proinflammatory resolvent with potent anti-inflammatory effects [6]. Numerous studies have shown that MaR1 can inhibit the release of proinflammatory factors and can promote macrophages to transform from the M1 phenotype to the M2 phenotype [7, 8]. Our previous studies have found that MaR1 can reduce the inflammatory response during brain I/R injury [9]. However, whether MaR1 can reduce the pyroptotic death of liver macrophages during liver I/R injury remains unclear, and we are strongly interested in whether MaR1 can reduce tissue damage by reducing liver macrophage pyroptosis during liver I/R injury.

In this study, we explored the protective effect of MaR1 on liver I/R injury and investigated the effect of MaR1 on liver macrophage pyroptosis and its specific mechanism.

## Materials and methods

### Animals and liver IR model

Male C57BL/6 mice aged 8 to 12 weeks and weighing 20–26 g were purchased from the Animal Experiment Center of Chongqing Medical University. RORa−/− and macrophage specific GSDMD−/− mice on the C57BL/6 genetic background were purchased from Shanghai Southern Model Biotechnology (Shanghai, China) and Shulaibao Biotech Company (Wuhan, China). Animals were allowed free access to regular rat chow and water and were housed on a 12 h light/dark cycle.

The liver I/R model was constructed according to previous studies [10, 11]. The mice were injected intraperitoneally with 50–80 mg/kg sodium pentobarbital and placed on the operating table after anesthesia. The abdominal cavity was opened, and the vascular pedicle of the left and middle liver lobes was exposed and clamped. One hour later, the blood vessels were reopened, and the abdominal incision was closed. Six hours after reperfusion, the mice were anesthetized again, and the liver tissue of the middle liver lobe was collected. All animal experiments were performed with approval from the Bioethics Committee of the Second Affiliated Hospital of Chongqing Medical University (No. 14 in 2023). Animal husbandry and experimental procedures were performed in full compliance with the United Kingdom Animal (Scientific Procedures) Act of 1986.

### Isolation of mouse liver primary macrophages

Mice were anesthetized, the abdominal cavity was opened, the portal vein and inferior vena cava were fully exposed, and the liver was lavaged with HBSS and 0.1% type IV collagenase. The liver was removed, cleaned with precooled HBSS and placed in preheated 0.1% type IV collagenase on a super clean table. The liver tissue was bluntly separated and prepared into suspension. The cell suspension was filtered through a 70 µm cell filter to prepare a single liver cell suspension. Primary liver macrophages were extracted by gradient centrifugation. Liver macrophages were then identified by cytometry.

### Cell culture, hypoxia/reoxygenation (H/R) model and cell transfection

RAW264.7 cells were obtained from the Cell Bank of the Chinese Academy of Sciences (Shanghai, China). Cells were cultured in Dulbecco’s Modified Eagle’s Medium (Gibco) with 20% fetal bovine serum (FBS). The H/R model was also established according to previous studies [10, 11]. Hypoxia preconditioning was performed in a tri-gas incubator with an N_2_ concentration of 95% and a CO_2_ concentration of 5% for 12 h in medium deprived of serum. Cells were then incubated with serum supplements under normal culture conditions for 2 h to induce reoxygenation injury.

For cell transfection, the lentivirus used to knock down and overexpress RORa was constructed by a commercial corporation (Oligobio, China). The sequence of RORa shRNA is shown in Additional file 1: Table S1.

### Measurements of ALT and AST levels

The levels of serum (or culture medium) ALT and AST were measured according to the manufacturer’s instructions (Solarbio, China). Briefly, the test solution and reagents 1–3 were added to 96-well plates separately in different orders. The standard curve was drawn using the standard solution. After mixing well for 15 min, the OD value was measured with a microplate reader at 505 nm.

### Measurement of NAD+/NADH

NAD+/NADH was detected using the WST-8 assay kit (Beyotime, China). This kit is based on a colorimetric reaction with WST-8 to detect the amount of NAD+/NADH in cells. The specific steps were as follows: NAD+/NADH extraction solution was added to the cells, and after 5–10 min, the supernatant was collected as the test sample. Then, NADH standard curve was prepared and set. Next, ethanol dehydrogenase working solution was added to the test samples and standard samples, followed by incubation and addition of the color development solution. Finally, the absorbance at 450 nm was measured to determine the NAD+/NADH levels.

### Mitochondrial membrane potential assay

Mitochondrial membrane potential (MMP) was assessed using a JC-1 MMP assay kit (Beyotime, China). After washing the cells with PBS, cell culture medium and JC-1 staining solution were added. The cells were incubated in the incubator for 20 min. After incubation, the cells were collected and washed with JC-1 staining buffer, and MMP was detected by flow cytometry.

### Mitochondrial permeability transition pore (MPTP)

The cells were washed twice with PBS, and calcein AM staining solution, fluorescence quenching solution or lonomycin control was added to the cells and incubated at 37 ℃ for 30 min according to the manufacturer’s instructions (Beyotime, China). After incubation, the culture medium was replaced with fresh prewarmed medium at 37 ℃ and incubated at 37 ℃ for 30 min. The detection buffer was then added to the cells, and the cells were observed under a fluorescence microscope.

### ROS assay

Cells were collected and suspended in DCFH-DA solution and incubated in a 37 °C cell incubator for 20 min. The cells were washed three times with serum-free cell culture solution. ROS were then detected by flow cytometry.

### ATP quantification assay

Cells were lysed using the reagent provided by the assay kit (Beyotime, China) and centrifuged at 12,000*g* at 4 ℃ for 5 min. The supernatants were collected for subsequent analysis. The ATP detection working solution was then added to the test solution and the standard solution. After mixing well, the RLU value was then measured using a luminometer.

### Mitochondrial respiratory chain complex activity

Mitochondrial respiratory chain complex I and III activity was measured by a commercial assay kit using the ultraviolet colorimetric method according to the manufacturer’s instructions (Sangon Biotech, China).

### Flow cytometry analysis of PI

Cells were washed twice with PBS and then resuspended. After staining with PI for 15 min at room temperature in the dark, the cells were analyzed by flow cytometry.

### ELISA assay

Mouse serum and cell culture medium were collected to measure IL-1β and IL-18 using commercially available ELISA kits (Beyotime, China) according to the manufacturer’s instructions.

### Western blot analysis

Western blotting was conducted as previously reported [12]. Briefly, cell proteins were lysed in RIPA buffer with phosphatase inhibitors and protease inhibitor (Sigma, USA). Antibodies for western blotting were purchased from Abcam (USA) and included the following: mouse anti-Caspase-1, mouse anti-Cleaved-caspase-1, mouse anti-GSDMD, mouse anti-GSDMD-N and mouse anti-RORa. Rabbit/mouse secondary antibodies were obtained from Beyotime Biotechnology (Shanghai, China). Proteins were separated by 10% SDS–polyacrylamide gels and transferred to PVDF membranes. After blocking, the PVDF membrane bands were incubated with specific and then secondary antibodies. The protein bands were detected using ECL developer and a Bio-Rad imaging system (Bio-Rad, USA). All experiments were repeated at least three times, and representative bands were chosen for this study.

### Quantitative reverse transcription-polymerase chain reaction (qRT‒PCR)

qRT–PCR was conducted as previously described [13]. Total RNA was extracted from cells using TRIzol reagent (Life Technologies, USA) according to the manufacturer’s instructions. Reverse transcription was conducted using a reverse transcription kit (Takara, Japan). qRT‒PCR was conducted using the quantitative SYBR Green PCR kit (Takara, Japan) according to the manufacturer’s instructions. All experiments were repeated at least three times. The primers used in the experiments are shown in Additional file 1: Table S2.

### Transcriptome analysis

The detection of the transcriptome was assisted by Biomarker Corporation (Biomarker, China). Total RNA was extracted using TRIzol reagent (Life Technologies, USA), and RNA concentration and purity were measured using a NanoDrop 2000 (Thermo Fisher Scientific, DE). RNA integrity was assessed using the RNA Nano 6000 assay kit and the Agilent Bioanalyzer 2100 system (Agilent Technologies, USA). Sequencing libraries were generated using the Hieff NGS Ultima Dual-mode mRNA Library Prep Kit for Illumina (Yeasen Biotechnology, China) following the manufacturer’s recommendations, and index codes were added to attribute sequences to each sample. The libraries were sequenced on an Illumina NovaSeq platform to generate 150 bp paired-end reads, according to the manufacturer’s instructions. The raw reads were further processed with a bioinformatic online platform (BMKCloud, www.biocloud.net). Quality control and further analysis were supported by the Biomarker Corporation (Biomarker, China).

### Nontargeted metabolomics analysis

Acetonitrile was added to the cells (cells > 1*10^6^), and the cells were broken by ultrasound in an ice bath. After centrifugation, the supernatant was absorbed and placed in a vacuum freeze-drying machine. After drying, the precipitate was redissolved in acetonitrile. After recentrifugation, the supernatant was taken for untargeted metabolomics analysis by a liquid mass spectrometer (AB SCIEX, USA).

### Statistical analysis

Data are presented as the mean ± standard deviation (SD) and were analyzed by GraphPad Prism 9.0. Student’s t test was used to compare differences between two groups. One-way analysis of variance (ANOVA) was used for multiple comparisons. Student’s t test or ANOVA followed by a post hoc test was used to determine statistically significant differences. P < 0.05 was considered significantly different.

## Results

### MaR1 dose-dependently attenuates liver I/R injury

**Fig. 1:**
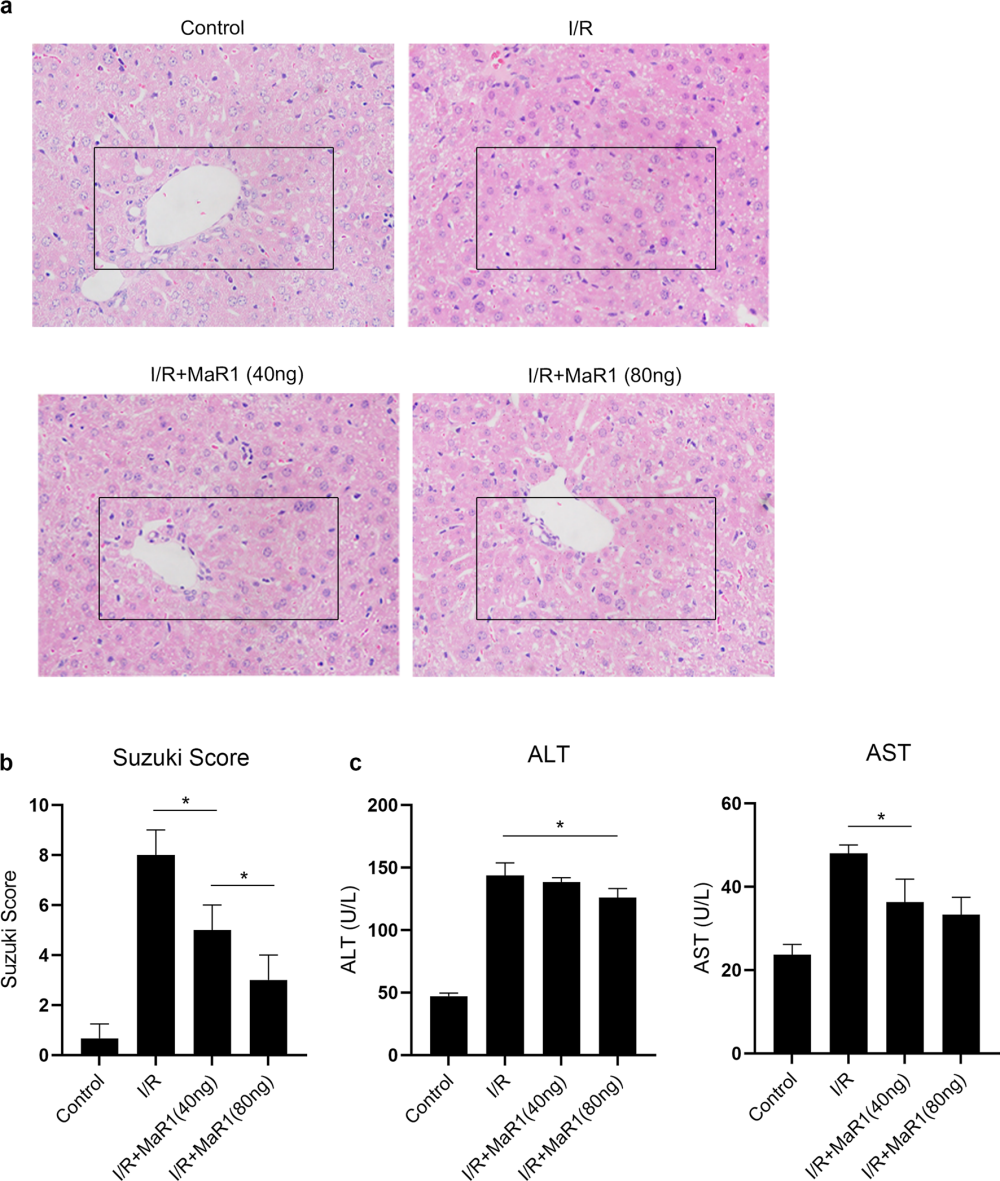
MaR1 attenuated liver I/R injury in mice. **a** Mice pretreated with different dosages of MaR1 were subjected to liver I/R injury. HE staining of the liver tissues showed that MaR1 attenuated liver I/R injury in a dose-dependent manner; **b** liver damage was assessed by Suzuki scores; **c** the serum levels of ALT and AST were evaluated (^*^P < 0.05 compared between groups)

We first analyzed the effects of MaR1 on mouse livers subjected to 60 min of warm ischemia followed by 6 h of reperfusion. Histologic observation showed that MaR1 dose-dependently decreased liver tissue injury. Liver lobular ballooning and necrosis were significantly decreased in mice pretreated with MaR1, and the Suzuki scores used to evaluate liver damage were lower (Fig. 1a, b). Meanwhile, the serum levels of ALT and AST were lower in mice pretreated with MaR1 (Fig. 1c). These results suggest that MaR1 effectively protected the liver from I/R injury.

### MaR1 alleviates liver I/R injury by reducing liver macrophage pyroptosis

**Fig. 2:**
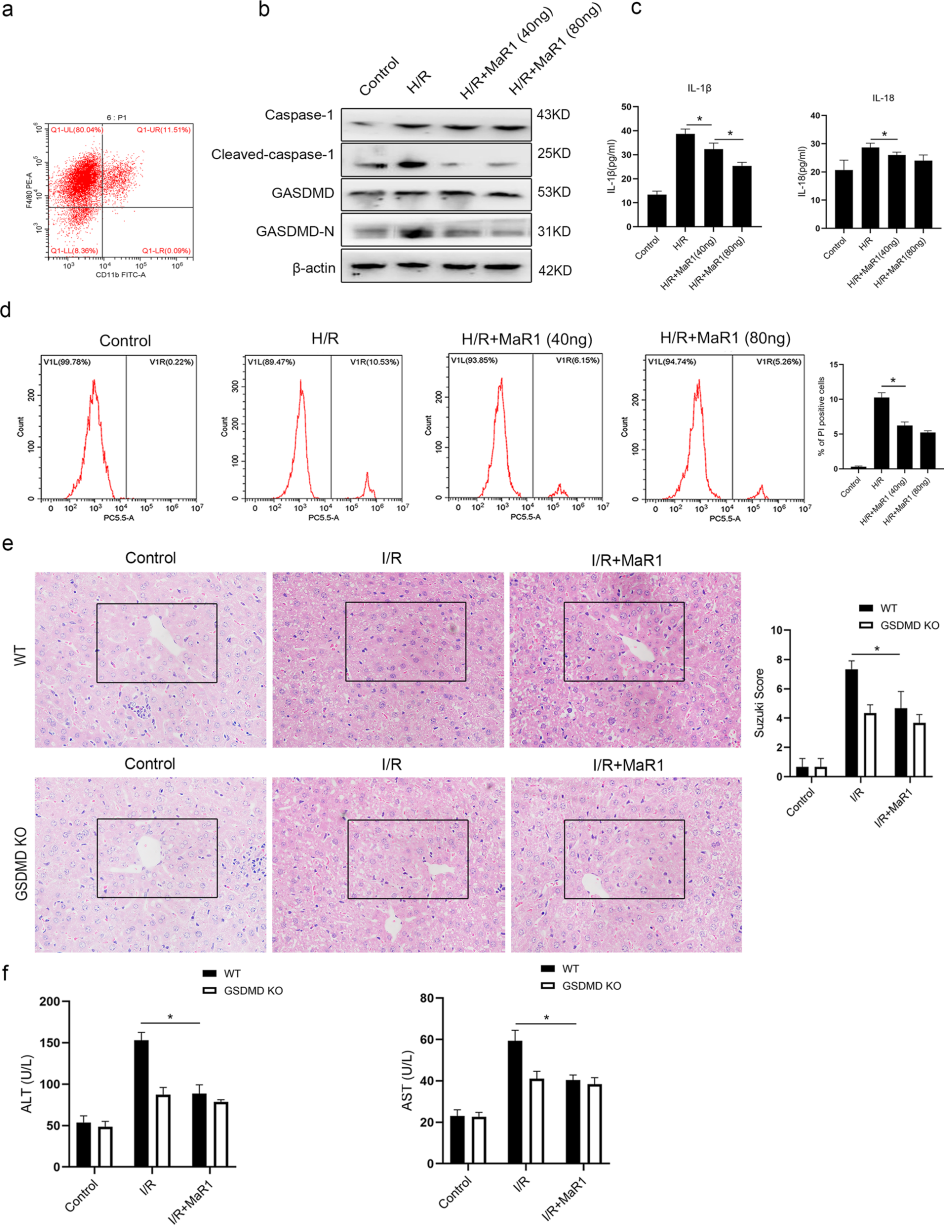
MaR1 alleviates liver I/R damage by reducing the pyroptotic death of liver macrophages. **a** Flow cytometry identification of M1-type liver macrophages; liver macrophages were pretreated with different dosages of MaR1 and then subjected to H/R injury. The protein expression levels of caspase-1, cleaved caspase-1, GSDMD and GSDMD-N in each group were evaluated by WB (**b**), the concentrations of IL-1β and IL-18 in the culture media were evaluated by ELISA (**c**), and the percentages of PI-positive cells in each group were detected by flow cytometry (**d**). GSDMD KO mice were constructed, and GSDMD KO mice and wild-type mice were pretreated with MaR1 and then subjected to liver I/R injury. HE staining of the liver tissues and Suzuki scores were used to evaluate liver damage in each group (**e**). Serum levels of ALT and AST were also evaluated (**f**) (*P < 0.05 compared between groups)

We then investigated whether MaR1 could decrease pyroptosis in liver macrophages. Primary hepatic macrophages were isolated (Fig. 2a), and cells were divided into control group, H/R group, H/R+MaR1 (40 ng) group and H/R+MaR1 (80 ng) group. Canonical pyroptosis, which relied on the cleavage and activation of Caspase-1 and GSDMD, in hepatic macrophages was induced (Fig. 2b–d). Next, we observed that MaR1 decreased the expression of cleaved caspase-1 and GSDMD-N in cells subjected to H/R injury (Fig. 2b). And MaR1 inhibited the release of IL-1β and IL-18 in liver macrophages (Fig. 2c). Also, MaR1 reduced the proportion of cells stained positive for PI (Fig. 2d). These results suggest that MaR1 could inhibit liver macrophage pyroptosis during H/R injury. We then studied whether inhibiting pyroptosis in liver macrophages is essential for MaR1 to alleviate liver I/R injury. We constructed macrophage-specific GSDMD knockout (GSDMD mKO) mice, the wild type and GSDMD mKO mice were divided into control group, I/R group and IR+MaR1 (80 ng) group. The results showed that compared to wild-type mice, GSDMD mKO mice experienced less severe tissue damage, with lower Suzuki scores and lower serum levels of ALT and AST (Fig. 2e, f). Furthermore, the protective effects of MaR1 were significantly decreased in GSDMD mKO mice (Fig. 2e, f). These results suggest that reducing pyroptosis in liver macrophages is an important mechanism by which MaR1 reduces liver I/R injury. We did not detect the occurrence of pyroptosis in hepatocytes because previous research reported that hepatocytes rarely undergo pyroptotic cell death [14].

### MaR1 reduces liver macrophage-mediated pyroptosis by protecting mitochondria

**Fig. 3:**
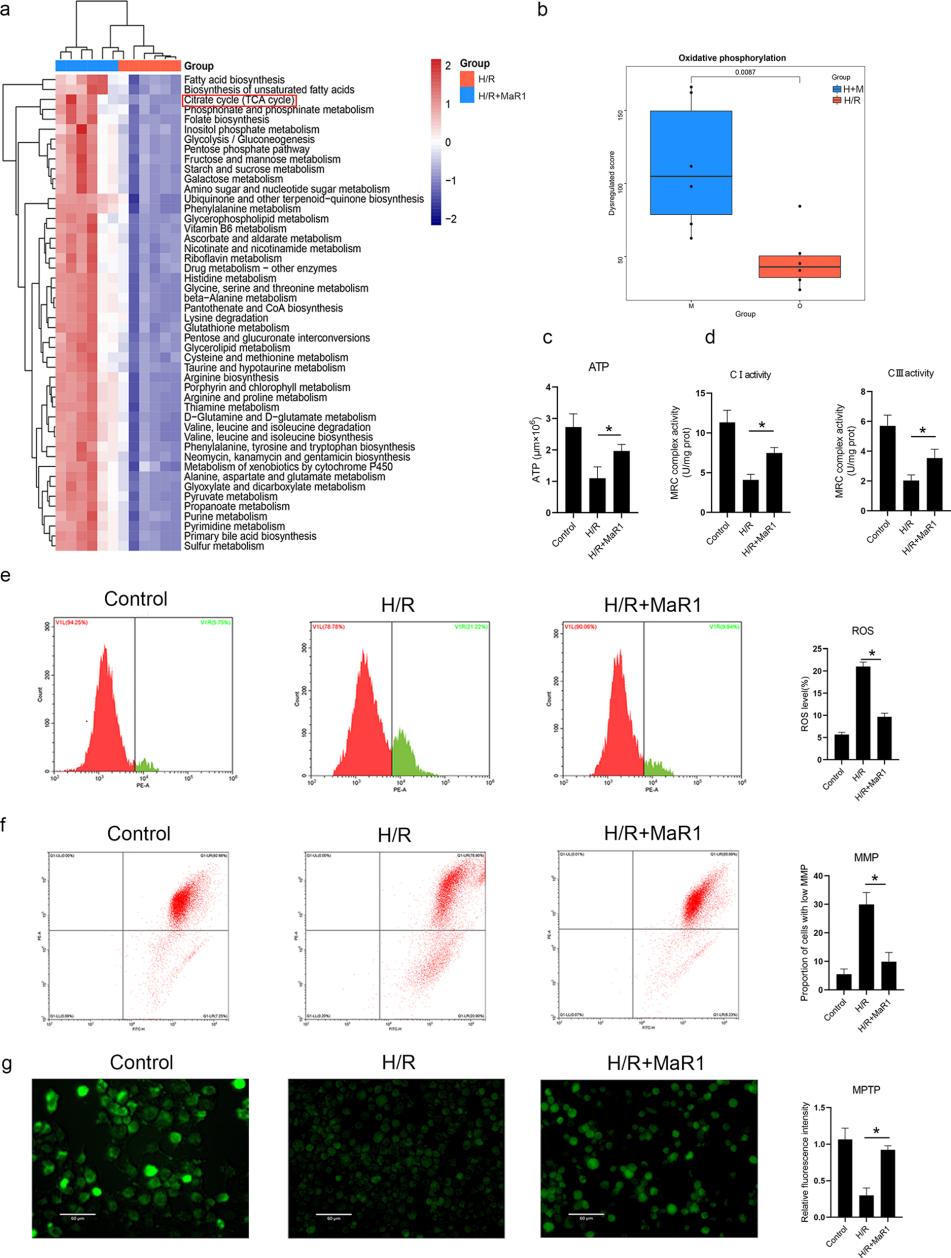
MaR1 protected mitochondria during H/R injury. Liver macrophages were subjected to H/R injury with or without MaR1 supplementation, and the metabolomics of cells were evaluated. The results showed that MaR1 increased cell oxidative phosphorylation (**a**, **b**). During H/R injury, MaR1 increased ATP production (**c**), increased MRC I and III activities (**d**), decreased ROS generation (**e**), stabilized the MMP (**f**), and decreased MPTP opening (**g**) (^*^P < 0.05 compared between gr

We then investigated the specific mechanism by which MaR1 reduces liver macrophage pyroptosis. Mitochondrial dysfunction is an important cause of cell pyroptosis during I/R injury. Mitochondrial dysfunction leads to increased intracellular ROS generation and decreased ATP synthesis. Intracellular ROS, as a classical DAMP, have been shown to cause cell pyroptosis. In addition, excessive ROS accumulation and ATP depletion eventually lead to MPTP opening, which can also induce cell pyroptosis [15]. Therefore, we studied the protective effects of MaR1 on mitochondria. Interestingly, we found that MaR1 promoted oxidative phosphorylation in mitochondria (Fig. 3a, b), increased the activities of mitochondrial respiratory chains, increased ATP production and reduced ROS generation (Fig. 3c–e). Cells treated with MaR1 were more stable in MMP and less prone to open MPTP (Fig. 3f, g). In addition, the protective effects of MaR1 on cell pyroptosis were significantly reduced in cells treated with hydrogen peroxide or CCCP (induce the opening of the MPTP) (Additional file 2: Fig. S1).

**Fig. 4:**
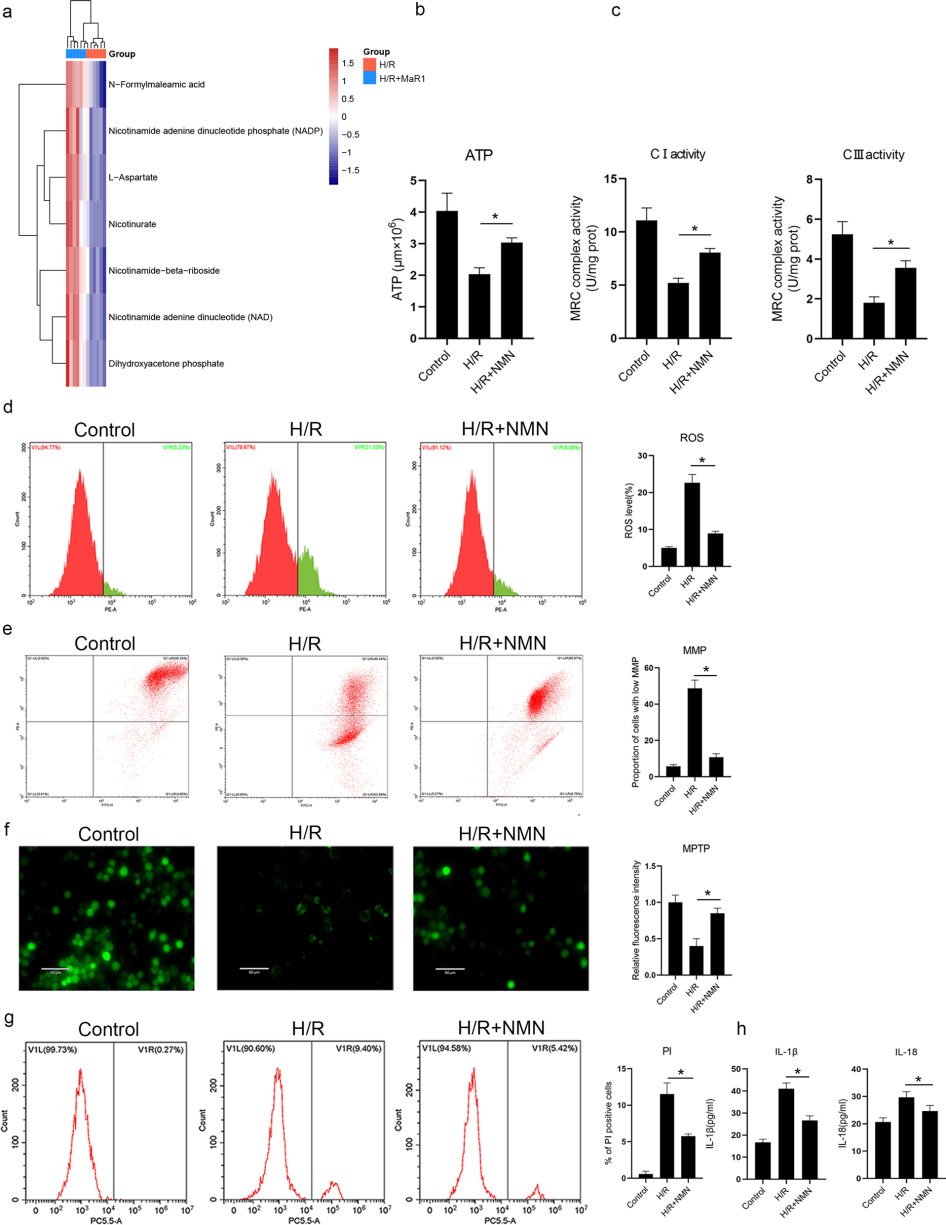
MaR1 increased the synthesis of NAD, and supplementation with NMN, the precursor of NADH, can play a protective role in mitochondria. Metabolomics results of liver macrophages showed that MaR1 increased nicotinate and nicotinamide metabolism (**a**); supplementation with NMN increased ATP production (**b**), increased MRC I and III activities (**c**), decreased ROS generation (**d**), stabilized the MMP (**e**), and decreased MPTP opening (**f**); and supplementation with NMN decreased cell death (**g**) and inhibited the release of IL-1β and IL-18 during H/R injury (**h**) (^*^P < 0.05 compared between groups)

Meanwhile, we found that MaR1 increased NAD synthesis (Fig. 4a). NAD is a major component of mitochondrial respiratory chain I and is also an important material for oxidative phosphorylation [16, 17]. NADH is also a classical antioxidant that reduces the damage caused by ROS. We found that supplementing cells with NMN (the precursor of NADH) led to increased ATP production, reduced ROS synthesis and a reduction in MPTP opening (Fig. 4b–f). More importantly, NMN supplementation significantly reduced the activation of Caspase-1 and GSDMD and reduced cell pyroptosis (Fig. 4g, h).

These results indicate that MaR1 has strong protective functions on mitochondria, which is an important mechanism by which MaR1 reduces cell pyroptosis.

### MaR1 protects mitochondria by activating RORa

**Fig. 5:**
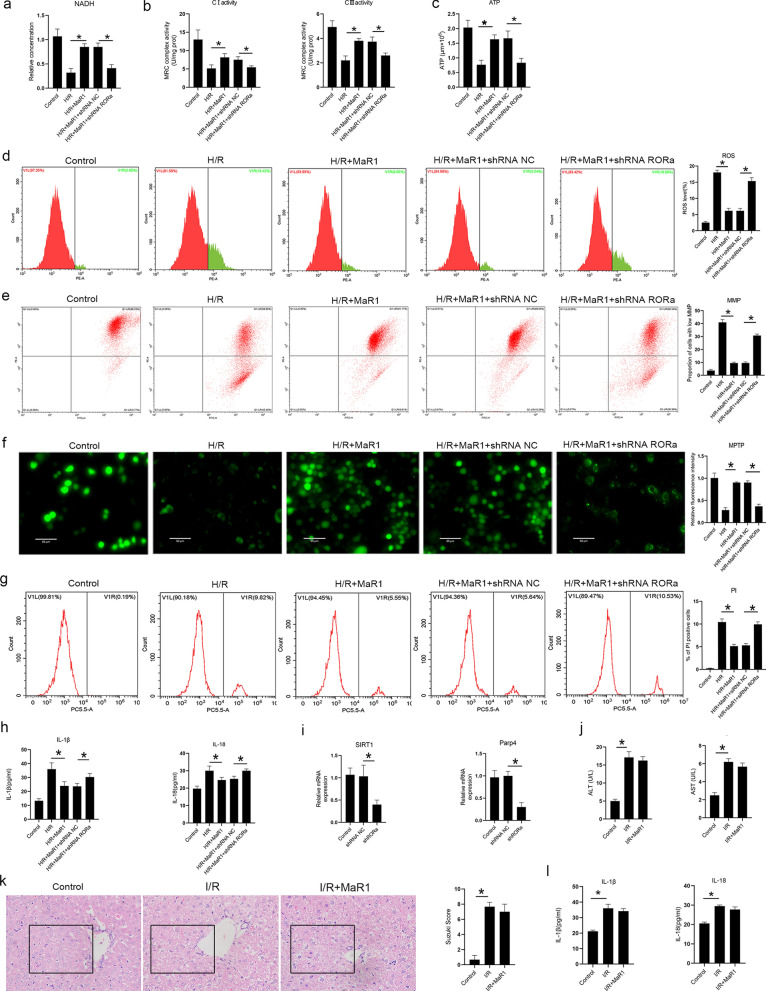
MaR1 protects mitochondria and reduces cell pyroptosis by activating RORa. RORa knockout cells were constructed, and the cells were subjected to H/R injury with or without MaR1 supplementation. The results showed that the synthesis of NADH was decreased in RORa knockout cells (**a**); the activities of MRC I and III (**b**) and the synthesis of ATP (**c**) were decreased in RORa knockout cells; the generation of ROS was elevated in RORa knockout cells (**d**); the MMP was impaired (**e**) and more MPTP were open in RORa knockout cells (**f**); PI flow cytometry showed cell death was increased in RORa knockout cells (**g**), ELISA showed the concentrations of IL-1β and IL-18 were increased in RORa knockout cells (**h**); qRT‒PCR showed the mRNA expressions of SIRT1 and Parp4 were decreased in RORa knockout cells (**i**); RORa KO mice were constructed, mice were subjected to liver I/R injury with or without the supplementation of MaR1. HE staining of the liver tissue and the Suzuki score (**j**), as well as the serum levels of ALT, AST, IL-1β and IL-18, were evaluated (**k**, **l**) (^*^P < 0.05 compared between groups)

RORa is a recently discovered nuclear receptor of MaR1 [18]. We thus knocked out RORa in liver macrophages to explore whether the protective effects of MaR1 depended on activating RORa. We found that RORa knockout significantly weakened the protective effects of MaR1 on mitochondria (Fig. 5a–f), and RORa knockout cells had more pyroptotic cell death during H/R injury (Fig. 5g, h). Furthermore, we found that knockout of RORa inhibited the expression of SIRT1 and Parp4 (Fig. 5i), which are not only key genes in the synthesis of NAD but also have strong protective effects on mitochondria.

To investigate the biological roles of RORa in vivo, we constructed RORa KO mice and subjected them to liver I/R injury. The results showed that the protective effects of MaR1 on the liver during I/R injury were significantly decreased in RORa KO mice (Fig. 5j, k), and their plasma levels of IL-1β and IL-18, which are related to cell pyroptosis, were significantly increased (Fig. 5l). These results indicated that MaR1 exerts a protective effect by activating RORa.

### MaR1 protects mitochondria by activating the PI3K-AKT pathway

**Fig. 6:**
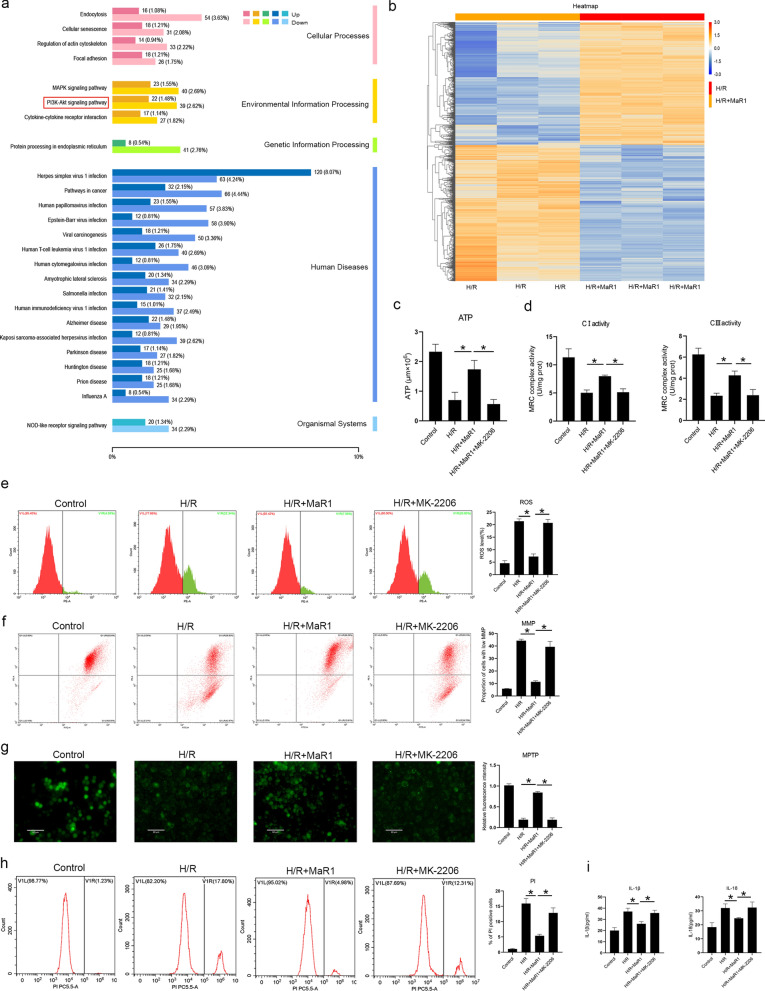
MaR1 protects mitochondria and reduces pyrosis by activating the PI3K-AKT pathway. Transcriptomic results showed that MaR1 activated the PI3K-AKT pathway in liver macrophages under H/R conditions (**a**, **b**); liver macrophages were divided into a control group, H/R group, H/R+MaR1 group and H/R+MaR1+MK-2206 group. The results showed that the AKT inhibitor MK-2206 decreased the protective effects of MaR1 on mitochondria and cell pyroptosis under H/R conditions. ATP synthesis (**c**), MRC I and III activities (**d**), ROS generation (**e**), MMP (**f**), MPTP opening (**g**), cell death (**h**) and IL-1β and IL-18 release (**i**) were evaluated in each group (^*^P < 0.05 compared between groups)

To explore whether MaR1 protects mitochondria through other mechanisms, we conducted transcriptomic studies on liver macrophages. We found that MaR1 activated the PI3K-AKT signaling pathway under H/R injury (Fig. 6a, b), which has a mitochondrial protective effect. We then used the AKT inhibitor MK-2206 to observe whether inhibition of the PI3K-AKT pathway would affect MaR1's protective effect on mitochondria. Interestingly, we found that the use of MK-2206 reduced mitochondrial ATP synthesis, increased ROS production, impaired mitochondrial membrane potential, and increased MPTP opening (Fig. 6c–g). In addition, the use of MK-2206 increased cell pyroptosis (Fig. 6h, i). Therefore, MaR1 also protects mitochondria and reduces pyroptotic cell death in liver macrophages by activating the PI3K-AKT signaling pathway.

## Discussion

This study found that MaR1 reduces liver I/R injury by inhibiting liver macrophage pyroptosis. The specific mechanism by which MaR1 inhibits macrophage pyroptosis involves the activation of its nuclear receptor RORa and the PI3K/AKT pathways, which promote mitochondrial oxidative phosphorylation, reduce ROS generation, decrease mitochondrial damage, and ultimately decrease cell pyroptosis.

Pyroptosis is a type of programmed cell death that was first described in 1992 [19], and this cell death process was named “pyroptosis” in 2001 [20]. Although pyroptotic cell death was described twenty years ago, how inflammatory caspase activation causes a lytic form of cell death was unclear until the identification of GSDMD as the executioner of pyroptosis [21, 22]. In recent years, the role of cell pyroptosis in various diseases has gradually been recognized. In infectious diseases, cell pyroptosis can help to confine viruses or bacteria in cell debris, and the subsequent immune response can also help eliminate foreign microorganisms [23, 24]. However, in the process of I/R injury, cell pyroptosis may cause an excessive inflammatory response and induce tissue damage [25]. In this study, liver macrophage pyroptosis was found to contribute to liver I/R injury, and inhibiting macrophage pyroptosis showed significant protective effects.

Mitochondria are one of the earliest and most severely damaged organelles during I/R injury [26], and we observed that MaR1 decreased cell pyroptosis by protecting mitochondria. MaR1 may also protect cells from other forms of programmed cell death, such as apoptosis and ferroptosis, by protecting mitochondria [27, 28]. However, cell pyroptosis is most likely to cause uncontrolled inflammatory damage [29]. From our point of view, this study was the first to find that MaR1 could promote NAD synthesis, enhance MRC activities, promote oxidative phosphorylation, reduce ROS generation, and reduce the opening of the MPTP. These results help us better understand the mechanisms by which MaR1 protects mitochondria.

Furthermore, we found that MaR1 protects mitochondria by activating its nuclear receptor RORa and by activating the PI3K/AKT signaling pathway. RORa is a nuclear transcription factor that regulates cell metabolism and cell stress reactions [30]. MaR1 could not only induce the expression of RORa but also increase its biological activities. The PI3K/AKT signaling pathway is also widely involved in cell survival, cell proliferation, protein synthesis, angiogenesis and other biological processes [31]. Our results indicate that activating RORa or the PI3K/AKT signaling pathway may be new ways to alleviate liver I/R injury. However, more studies are needed to verify this hypothesis.

This study extensively explored the effects of MaR1 on mitochondrial damage and cell pyroptosis in liver macrophages, and found that attenuating macrophage pyroptosis can reduce I/R injury in the liver. However, the available results do not fully demonstrate that MaR1 primarily alleviates liver I/R injury by reducing macrophage pyroptosis. In fact, MaR1 may also reduce liver tissue inflammation through mechanisms such as decreasing the release of inflammatory mediators and promoting macrophage polarization from M1 to M2. These mechanisms have not been discussed in this study. But our research has proposed a new mechanism by which MaR1 alleviates liver I/R injury, and indicates potential new methods for reducing liver I/R injury—such as inhibiting macrophage pyroptosis, which opens up avenues for the development of novel drugs to reduce liver I/R injury.

In conclusion, this study found that MaR1 reduced the pyroptotic death of liver macrophages by protecting mitochondria and thus reduced liver I/R injury. These results provide us with a better understanding of the role of the pyroptotic death of liver macrophages in liver I/R injury and help us find new ways to reduce liver I/R injury.

## Supplementary Information

**Additional file 1: Table S1.** Sequences of shRNA. **Table S2. **Primer sequences for qRT-PCR in study.

**Additional file 2**:** Figure S1**. ROS and MPTP opening may induce the pyroptotic death of liver macrophages. Liver macrophages were divided into the control group, H/R group, H/R+MaR1 group, H/R+MaR1+H_2_O_2_ group and H/R+MaR1+CCCP group. The expression of GSDMD and GSDMD-N (**a**) and the release of IL-1β and IL-18 (**b**) were evaluated.

## Data Availability

The datasets used and/or analyzed during the current study are available from the corresponding author upon reasonable request.

## Abbreviations

MaR1: Maresin1
ROS: Reactive oxygen species
RORa: Retinoic acid–related orphan receptor α
SIRT1: Sirtuin 1
I/R: Ischemia‒reperfusion
MPTP: Mitochondrial permeability transition pore
MMP: Mitochondrial membrane potential
GSDMD: GasderminD
GSDMD-N: N-terminal cleavage product of GSDMD
