# Supplementary material for: Maresin1 alleviates liver ischemia/reperfusion injury by reducing liver macrophage pyroptosis
Source: J Transl Med. 2023 Jul 16;21:472. doi: 10.1186/s12967-023-04327-9 (PMC10351145; doi:10.1186/s12967-023-04327-9)
Supplement: Supplementary file 1 — Additional file 1: Table S1. Sequences of shRNA. Table S2. Primer sequences for qRT-PCR in study. [file 12967_2023_4327_MOESM1_ESM.docx]

**Table S1. Sequences of shRNA**

| Target | Sequence |
| --- | --- |
| shRORa | 5’- GCAGAGAGACAGCTTGTACGC -3’ |
| shRNA-NC | 5’-CCATTCACTTGATCCACATGCGTGTAGCA-3’ |

**Table S2. Primer sequences for qRT-PCR in study.**

| Target | Sequence |
| --- | --- |
| SIRT1 | Sense: 5’- GCTGACGACTTCGACGACG -3’ |
|  | Anti-sense: 5’- TCGGTCAACAGGAGGTTGTCT -3’ |
| Parp4 | Sense: 5’- TCATACCACCTAAGTTGGGTCC -3’ |
|  | Anti-sense: 5’- AGCAAAGCTACCTGAGAAAGC -3’ |
| GAPDH | Sense: 5’- GGAGCGAGATCCCTCCAAAAT -3’ |
|  | Anti-sense: 5’- GGCTGTTGTCATACTTCTCATGG −3’ |
