# Supplementary material for: Maresin1 alleviates liver ischemia/reperfusion injury by reducing liver macrophage pyroptosis
Source: J Transl Med. 2023 Jul 16;21:472. doi: 10.1186/s12967-023-04327-9 (PMC10351145; doi:10.1186/s12967-023-04327-9)
Supplement: Supplementary file 2 — Additional file 2: Figure S1. ROS and MPTP opening may induce the pyroptotic death of liver macrophages. Liver macrophages were divided into the control group, H/R group, H/R+MaR1 group, H/R+MaR1+H2O2 group and H/R+MaR1+CCCP group. The expression of GSDMD and GSDMD-N (a) and the release of IL-1β and IL-18 (b) were evaluated. [file 12967_2023_4327_MOESM2_ESM.docx]

**
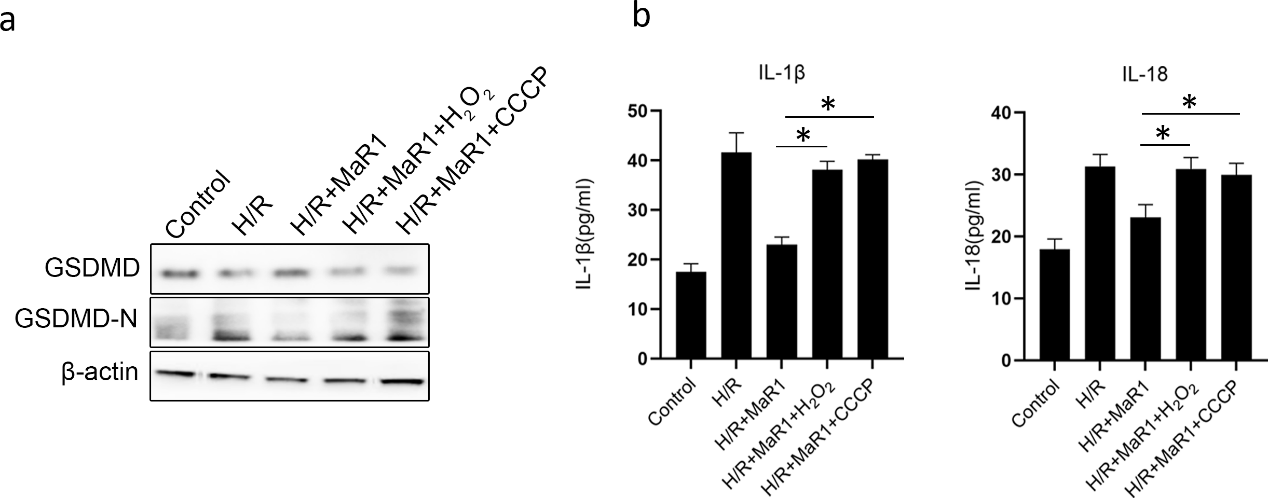
**

**Figure S1**. **ROS and MPTP opening may induce the pyroptotic death of liver macrophages**. Liver macrophages were divided into the control group, H/R group, H/R+MaR1 group, H/R+MaR1+H_2_O_2_ group and H/R+MaR1+CCCP group. The expression of GSDMD and GSDMD-N (**a**) and the release of IL-1β and IL-18 (**b**) were evaluated.
